# Tracking early cognitive decline in preclinical AD with brain MRI similarity

**DOI:** 10.1002/alz.71170

**Published:** 2026-03-18

**Authors:** Jiawei Sun, Blanca Zufiria‐Gerbolés, Massimiliano Passaretti, Giovanni Volpe, Mite Mijalkov, Joana B. Pereira, for the Alzheimer's Disease Neuroimaging Initiative

**Affiliations:** ^1^ Department of Clinical Neuroscience Division of Neuro Karolinska Institutet Stockholm Sweden; ^2^ Department of Physics Goteborg University Goteborg Sweden; ^3^ Science for Lifesciences Laboratory Department of Physics Goteborg University Goteborg Sweden

**Keywords:** Alzheimer's disease, brain similarity, cognitive decline, cortical thickness, diagnostic conversion, fluid biomarkers, preclinical Alzheimer's disease, structural magnetic resonance imaging

## Abstract

**INTRODUCTION:**

Early detection of neuroanatomical changes in preclinical Alzheimer's disease (AD) is critical for timely intervention. However, conventional magnetic resonance imaging (MRI) and fluid biomarkers often lack sensitivity to subtle structural alterations in early disease stages.

**METHODS:**

To identify early brain alterations, we applied a perturbation‐based brain similarity approach to cognitively normal participants from Alzheimer's Disease Neuroimaging Initiative (ADNI) and Open Access Series of Imaging Studies (OASIS), stratified by amyloid status. We evaluated its predictive performance for cognition and diagnostic conversion against cortical thickness, volumetric MRI, and fluid biomarkers.

**RESULTS:**

In both cohorts, brain similarity consistently outperformed other biomarkers across cognitive domains and amyloid groups. It also achieved superior accuracy in predicting clinical conversion and exhibited associations with cytoarchitectural organization.

**DISCUSSION:**

These findings highlight brain similarity as a sensitive marker of early neuroanatomical disruption in AD. Its ability to detect subtle structural changes before overt atrophy underscores its potential for early disease monitoring and treatment assessment in preclinical AD trials.

**Highlights:**

- Brain similarity captures early brain changes in preclinical Alzheimer's disease (AD).
- Brain similarity outperforms conventional biomarkers such as cortical thickness, volume measures, and fluid biomarkers in predicting cognitive decline.
- Brain similarity predicts conversion to mild cognitive impairment and AD more accurately than traditional imaging markers, and its predictive performance is further improved when combined with fluid biomarkers.
- Brain similarity captures structural disruptions associated with cortical layer II of the cytoarchitectonic lamina of human neocortex.

## 1 BACKGROUND

Alzheimer's disease (AD) begins long before the onset of cognitive symptoms, with pathological changes emerging more than a decade prior to diagnosis.^1^ These changes are thought to be driven by the accumulation of amyloid beta (Aβ) into fibrillar plaques, initiating a cascade of neurodegenerative events, including tau hyperphosphorylation (p‐tau) and synaptic dysfunction.^2^, ^3^ Recent estimates indicate that ≈ 20% to 40% of cognitively unimpaired older adults exhibit elevated Aβ deposition, placing them at increased risk for cognitive decline.^4^ Therefore, identifying reliable biomarkers that can predict disease progression during this preclinical stage is crucial for developing effective early interventions.^5^

Recognizing the need for early detection, recent clinical trials, such as the AHEAD 3‐45 study, have shifted focus to the preclinical phase of AD, in contrast to earlier studies targeting symptomatic individuals.^6^ These trials evaluate the effects of anti‐amyloid therapies in cognitively unimpaired individuals with elevated or intermediate levels of Aβ pathology, aiming to determine whether reducing Aβ accumulation can delay or prevent progression in those already Aβ positive or have a high risk of becoming positive. However, clinical symptoms are often minimal or absent at this early stage, complicating the evaluation of intervention outcomes using conventional measures. A key challenge in such trials is the lack of sensitive biomarkers to predict subtle clinical progression and evaluate treatment efficacy.

In current AD clinical trials, most biomarkers unrelated to Aβ pathology are obtained from structural magnetic resonance imaging (MRI) metrics or fluid‐based assays.^7^, ^8^ While these measures are effective in later disease stages, they lack the sensitivity to capture early alterations in preclinical AD.^5^ Structural MRI measures such as hippocampal, ventricular, and whole‐brain volumes are commonly used in clinical AD trials but may not detect changes in cognitively unimpaired individuals with elevated Aβ, who often exhibit preserved brain structure, with atrophy occurring later in the course of AD.^9^, ^10^ Similarly, cerebrospinal fluid (CSF) and plasma biomarkers, such as p‐tau181, show substantial variability among Aβ‐positive individuals, with some displaying abnormal levels while others are within the normal range.^11^, ^12^ Neurodegeneration markers, including neurofilament light chain (NfL) and total tau (t‐tau), become abnormal only after significant cognitive decline has begun—typically in the mild cognitive impairment (MCI) or dementia stages.^13^, ^14^ These findings highlight the need for sensitive markers to detect subtle changes in early disease stages.

Advances in MRI research suggest that brain similarity offers a promising approach to detect earlier changes missed by atrophy‐based measures.^15^, ^16^, ^17^, ^18^ This method assesses the relationships between regional morphological characteristics, which reflect cytoarchitecture, as regions that covary in their morphology often belong to the same cortical layer.^15^ Notably, AD is associated with early alterations in cortical layer II,^19^, ^20^ suggesting that brain similarity could detect related cytoarchitectural disruptions.

To test this hypothesis, we computed brain MRI similarity by assessing regional covariance in cortical thickness among cognitively normal individuals with significantly elevated Aβ pathology at baseline or follow‐up.^17^, ^21^ We applied a perturbation‐based method to construct individualized brain similarity networks for each subject.^17^, ^22^ This approach generates whole‐brain similarity profiles that reflect deviations from a normative reference group and is conceptually related to normative modeling frameworks in precision medicine.^22^ We then compared the performance of brain MRI similarity to commonly used clinical and preclinical AD trial measures, including regional volume, cortical thickness, and plasma/CSF biomarkers in predicting cognitive decline and diagnostic conversion.

Our results demonstrate that brain MRI similarity outperforms currently used biomarkers in detecting cognitive changes among Aβ‐positive individuals or those who later convert. Across two independent cohorts, this approach provided more accurate predictions of decline in global cognition, preclinical cognitive markers, and specific cognitive domains compared to currently used measures. It also predicted conversion to MCI and dementia due to AD. Given that T1‐weighted MRI scans are routinely acquired in amyloid positron emission tomography (PET)‐based preclinical trials, our findings suggest that brain similarity could serve as an additional, cost‐effective, and sensitive outcome for evaluating treatment efficacy and guiding early interventions.

## 2 METHODS

### 2.1 Participants

This study included 185 individuals from the Alzheimer's Disease Neuroimaging Initiative (ADNI; adni.loni.usc.edu), a longitudinal and multicenter cohort designed to identify biomarkers and track the progression of AD.^23^ For this study, we only included individuals who were cognitively normal at baseline and had longitudinal cognitive assessments, structural MRI scans, and 18F‐florbetapir amyloid PET imaging. All participants were required to have a Clinical Dementia Rating (CDR)^24^ score of 0 and a Mini‐Mental State Examination (MMSE)^25^ score between 27 and 30, and not meet the criteria for MCI or AD dementia at baseline. Individuals with a history of significant neurological disorders (e.g., stroke, Parkinson's disease) or primary psychiatric conditions were excluded.

Cognitive performance was assessed using standardized neuropsychological tests across multiple cognitive domains. Executive function, attention, and language were measured using the Trail Making Test Part B (TMT‐B),^26^ Trail Making Test Part A (TMT‐A),^26^ and the Boston Naming Test (BNT),^27^ respectively. Memory was assessed using the Alzheimer's Disease Assessment Scale‐Cognitive Subscale Delayed Recall (ADASQ4),^28^ and visuospatial abilities were evaluated using the Clock Drawing Test.^29^ In addition, we also included activities of daily living (ADL)^30^ and a modified version of the Preclinical Alzheimer Cognitive Composite (PACC),^31^ which is commonly used in preclinical trials of AD.^32^ The PACC was constructed by standardizing and averaging scores from ADASQ4, Logical Memory Delayed Recall Total Score (LMDRT),^33^ TMT‐B, and MMSE.

To test the reproducibility of our findings, we included a replication sample of 341 individuals from the Open Access Series of Imaging Studies (OASIS; https://sites.wustl.edu/oasisbrains), a publicly available neuroimaging dataset designed to support research on brain aging and neurodegenerative diseases.^34^ The inclusion criteria for OASIS were identical to those used in ADNI.

In the OASIS cohort, executive function, attention, and language were evaluated using TMT‐B, TMT‐A, and BNT, respectively. Memory was assessed using LMDRT in OASIS. Visuospatial abilities were not assessed in the dataset. In the OASIS cohort, we constructed a modified PACC using LMDRT, TMT‐B, and MMSE, consistent with the PACC framework capturing three core cognitive domains relevant to preclinical AD: episodic memory, executive function, and global cognition.^31^

**RESEARCH IN CONTEXT**

1. **Systematic review**: We reviewed the literature on fluid biomarkers and structural magnetic resonance imaging (MRI) measures used in preclinical Alzheimer's disease (AD). Prior studies indicate that traditional biomarkers show limited sensitivity for detecting very early, preclinical changes.
2. **Interpretation**: Our study demonstrates that subject‐level brain similarity networks, derived from structural MRI with the proposed method, outperform conventional biomarkers in predicting cognitive decline and diagnostic conversion in amyloid beta–positive individuals. These results suggest brain similarity is a sensitive and accessible biomarker that captures early system‐level structural disruptions, particularly within cortical layer II.
3. **Future directions**: Future studies should validate brain similarity as a biomarker in preclinical AD, with emphasis on the following directions: (a) replication in larger cohorts of preclinical AD participants, (b) assessment of its potential utility as an outcome measure in preclinical AD trials, and (c) evaluation of its sensitivity to treatment effects, including longitudinal changes in response to therapeutic intervention.

To standardize measurements and facilitate comparisons across participants, all cognitive test scores were converted to *z* scores based on the mean and standard deviation of the respective cohort. In addition, for ADASQ4, TMT‐A, and TMT‐B tests, scores were inverted so that higher scores indicated better cognitive function.

Participants were categorized into two groups based on baseline and longitudinal Aβ status: Aβ negative at baseline who became Aβ positive at follow‐up (incident Aβ positive) and Aβ positive at baseline and follow‐up (elevated Aβ positive). All analyses were performed separately for these two groups. Subjects who were Aβ negative both at baseline and longitudinally were only used to build the reference structural covariance matrix.

To ensure temporal consistency across imaging and cognitive measures in the longitudinal analyses, we applied stringent inclusion criteria. Specifically, for each included observation, cognitive assessments, diagnostic information, and amyloid PET imaging were required to be acquired within a 6 month window centered on the date of the MRI scan. This ensured that data were temporally aligned, minimizing potential bias due to timing differences. Details regarding subject selection procedures are shown in Figure S1 in supporting information.

To further examine the generalizability of brain similarity, we also analyzed a cohort of healthy controls from the Parkinson's Progression Markers Initiative (PPMI; https://www.ppmi‐info.org/).^35^ PPMI is a large longitudinal study focused on biomarker discovery in Parkinson's disease, and its healthy control cohort consists of rigorously screened individuals without neurological disorders. To investigate whether brain similarity measures can predict longitudinal cognitive changes in cognitively normal individuals without Aβ pathology but at increased genetic risk due to apolipoprotein E (*APOE*) ε4, we selected cognitively normal, Aβ‐negative participants. The sample included 98 *APOE* ε4 non‐carriers (reference group) and 27 *APOE* ε4 carriers, in whom we tested whether baseline brain similarity or cortical thickness predicted subsequent cognitive change. Longitudinal cognition was assessed across six domains (global cognition, visuospatial ability, memory, executive function, language, and attention).^36^ Full details of the dataset and analytical procedures are provided in Section S1 and Table S1 in supporting information.

The regional ethical committees of both cohorts approved the study and written informed consent was obtained from all participants according to the Declaration of Helsinki.

### 2.2 Image preprocessing

Structural MRI and PET images were obtained from the ADNI and OASIS cohorts as preprocessed data having already undergone standardized image processing and quality control by the respective data repositories.^23^, ^34^, ^37^ No additional preprocessing was performed prior to analysis. Further details on the preprocessing procedures are provided in sections 2.2.1 and 2.2.2.

#### 2.2.1 MRI acquisition and preprocessing

Structural MRI scans in ADNI were acquired using a 3T MRI scanner with a T1‐weighted magnetization‐prepared rapid gradient echo (MPRAGE) sequence. The acquisition parameters were as follows: repetition time (TR) = 2300 ms, echo time (TE) = 2.9 ms, inversion time (TI) = 900 ms, flip angle = 9°, and slice thickness = 1.2 mm. The field of view (FOV) was 208 × 240 × 256 mm, with a voxel size of 1 × 1 × 1 mm^3^.

Structural MRI data were preprocessed using FreeSurfer version 5.1,^38^ using the standard recon‐all pipeline, which includes motion correction, intensity normalization, skull stripping, linear and non‐linear registration, subcortical segmentation, cortical surface reconstruction, and parcellation based on gyral and sulcal anatomy. Quality control (QC) was conducted using standardized protocols and only participants who passed QC were included in the analysis.^39^ Volumetric measures, including average hippocampal, total gray matter, and ventricular volume were obtained from the preprocessed images, as these are commonly used biomarkers in AD clinical trials.^40^, ^41^, ^42^ For cortical thickness analyses, values were extracted from all 68 cortical regions defined by the Desikan–Killiany atlas.^39^

In the OASIS dataset, structural MRI scans were also acquired using a 3T scanner with a T1‐weighted MPRAGE sequence. The acquisition parameters were: TR = 2300 ms, TE = 2.9 ms, TI = 900 ms, flip angle = 9°, and slice thickness = 1.2 mm with no interslice gap. The matrix size was 240 × 240, with a voxel size of 1.20 × 1.05 × 1.05 mm^3^. MRI preprocessing was performed using FreeSurfer version 5.0,^38^ following the same recon‐all pipeline as in ADNI. For QC, visual inspection was carried out, and manual corrections were applied when segmentation errors exceeded predefined thresholds.^34^ Similar to ADNI, both volumetric and cortical thickness measures were extracted for further analysis.

#### 2.2.2 PET acquisition and preprocessing

In ADNI, amyloid PET imaging was performed using [18F]‐florbetapir (AV45) to assess Aβ deposition. PET images were acquired from the Laboratory of Neuroimaging (LONI) in a fully preprocessed format, including frame realignment, head orientation standardization, and uniform voxel size resampling. The closest available T1‐weighted MRI scan was used for co‐registration to improve anatomical alignment. The amyloid burden was quantified using the cortical summary region, which included the frontal, anterior, and posterior cingulate, lateral parietal, and lateral temporal cortices, defined based on Desikan–Killiany atlas. Images were intensity normalized using a composite reference region (whole cerebellum, brainstem, and eroded subcortical white matter) for longitudinal assessments to reduce inter‐scan variability.^37^ Aβ positivity was classified based on a previously validated standardized uptake value ratio threshold of 1.11, which was derived from the upper 95% confidence interval of a young cognitively normal reference group.^43^

In OASIS, amyloid PET imaging was performed using [11C]‐Pittsburgh compound‐B (PiB) and AV45 to assess Aβ deposition. PET images were acquired using Siemens ECAT HR+ PET and Biograph PET/MR scanners, following standardized acquisition protocols.^34^ PiB scans were acquired dynamically over 60 minutes, starting from injection time, while AV45 scans were acquired 50 to 70 minutes post‐injection. Image preprocessing was conducted using the PET Unified Pipeline,^44^ which included motion correction, spatial normalization to 8 mm resolution, and co‐registration to T1‐weighted MRI scans. Partial volume correction was applied using the regional spread function method to account for spillover effects.^44^ The amyloid burden was quantified using Centiloid scaling, facilitating cross‐tracer comparisons. Aβ positivity was classified based on OASIS3‐provided Centiloid thresholds, with values > 16.4 for PiB and 20.6 for AV45.^34^

### 2.3 CSF and plasma biomarker measurements

Participants from ADNI underwent lumbar puncture for CSF biomarker analyses and provided plasma samples. Plasma NfL and plasma p‐tau181)were available for 71 participants, while CSF p‐tau181 and t‐tau were available for 60 participants. Due to the smaller number of participants with available biomarker data, all biomarker‐related analyses were conducted in this subset (see Table 1 for details). Biomarker values were log transformed before analysis to account for skewed distributions and improve statistical interpretability.

**TABLE 1:** Characteristics of the individuals from the ADNI and OASIS cohorts.

	ADNI			OASIS		
Cohort Group	Stable Aβ negative	Incident Aβ positive	Elevated Aβ positive	Stable Aβ negative	Incident Aβ positive	Elevated Aβ positive
*N*	114	26	45	243	36	71
Age, years	71.5 (8.87)	69.8 (7.43)	75 (7.9)**	65.1 (13.68)	67.8 (7.52)*	71.5 (7.5)***
Sex, male/female	59 (55)	18 (8)	26 (19)	90 (153)	14 (22)	31 (40)
Education, years	17 (3)	17.5 (4)	16 (4)	16 (4)	16 (4.5)	16 (4.75)
Imaging follow‐up, years	2 (1.08)	2 (1.02)	2 (1.08)	4.4 (3.5)	7.9 (3.99)	4 (3.33)
**Cognitive performance**						
ADASQ4	2 (2)	2 (2)	3 (3)**	–	–	–
LMDRT	–	–	–	15 (6)	13 (5)	13 (6)
TMA	30 (12)	29 (15)	35 (14.5)**	29 (14)	28.5 (12)	30 (18)
TMB	70.5 (36)	61 (27)	80 (38)*	67 (37)	72.5 (31.5)	69 (41.5)
BNT	29 (3)	29 (2)	29 (3)	56 (4)	56 (6)	56 (5.5)
Clock	5 (0)	5 (1)	5 (1)	–	–	–
Copy	5 (0)	5 (0)	5 (0)	–	–	–
PACC	0.9 (3.75)	1.6 (2.52)	−1.3 (3.4)**	1 (2.4)	0.7 (2.52)	0.4 (3.04)
ADL	0 (0)	0 (0)	0 (0)	0.6 (0.11)	0.6 (0.9)	0.6 (0.7)
**CSF biomarkers**						
N	114	26	45	–	–	–
NfL, pg/mL	30 (16.05)	31.8 (15.9)	39.8 (19.47)***	–	–	–
P‐tau181 pg/Ml	12 (7.7)	14.1 (8.12)*	19 (8.18)***	–	–	–
**Plasma biomarkers**						
*n*	91	21	39	–	–	–
P‐tau181, pg/mL	17.2 (7.34)	25.4 (17.8)**	27.1 (15.85)***	–	–	–
T‐tau, pg/mL	195.7 (76.4)	281.3 (170.4)**	265.6 (144.12)***	–	–	–

*Notes*: All values represent baseline data. Data are presented as median (interquartile range), except for sex, which is shown as the number of males and females. Significant differences are reported relative to the Aβ‐negative group within each cohort (ADNI or OASIS). Continuous variables were assessed using the Mann–Whitney *U* test, and categorical variables using the chi‐squared test. Significance levels: *p* < 0.05 (*), *p* < 0.01 (**), *p* < 0.001 (***). “—” indicates that the data were not collected or not applicable for this group.

Abbreviations: Aβ, amyloid beta; ADASQ4, Delayed Word Recall item from the Alzheimer's Disease Assessment Scale‐Cognitive Subscale; ADL, Activities of Daily Living Questionnaire; ADNI, Alzheimer's Disease Neuroimaging Initiative; BNT, Boston Naming Test; CSF, cerebrospinal fluid; LMDRT, Logical Memory Delayed Recall Test; NfL, neurofilament light chain; OASIS, Open Access Series of Imaging Studies; PACC, Preclinical Alzheimer Cognitive Composite; p‐tau, phosphorylated tau; TMA/TMB, Trail Making Test Part A/B; t‐tau, total tau.

CSF samples were collected via lumbar puncture, typically performed in the morning after an overnight fast. The procedure followed standardized ADNI protocols, ensuring consistency across different collection sites. CSF p‐tau181 and t‐tau were quantified using the electrochemiluminescence immunoassay (ECLIA) on the Roche Elecsys system, a highly automated and standardized platform.

Plasma samples were collected through venipuncture. Plasma p‐tau181 and NfL concentrations were measured using the Single Molecule Array (Simoa) technology, an ultra‐sensitive digital immunoassay method developed by the Clinical Neurochemistry Laboratory at the University of Gothenburg. The p‐tau181 assay used a pair of monoclonal antibodies (tau12 and AT270) to target N‐terminal to mid‐domain epitopes of the protein. For the NfL assay, a combination of monoclonal antibodies was used in conjunction with purified bovine NfL as the calibrator.

Further details regarding biomarker acquisition protocols are available at the ADNI website (http://adni.loni.usc.edu). Biomarker data were not available in the OASIS cohort.

### 2.4 Construction of individual brain similarity matrices

**FIGURE 1:**
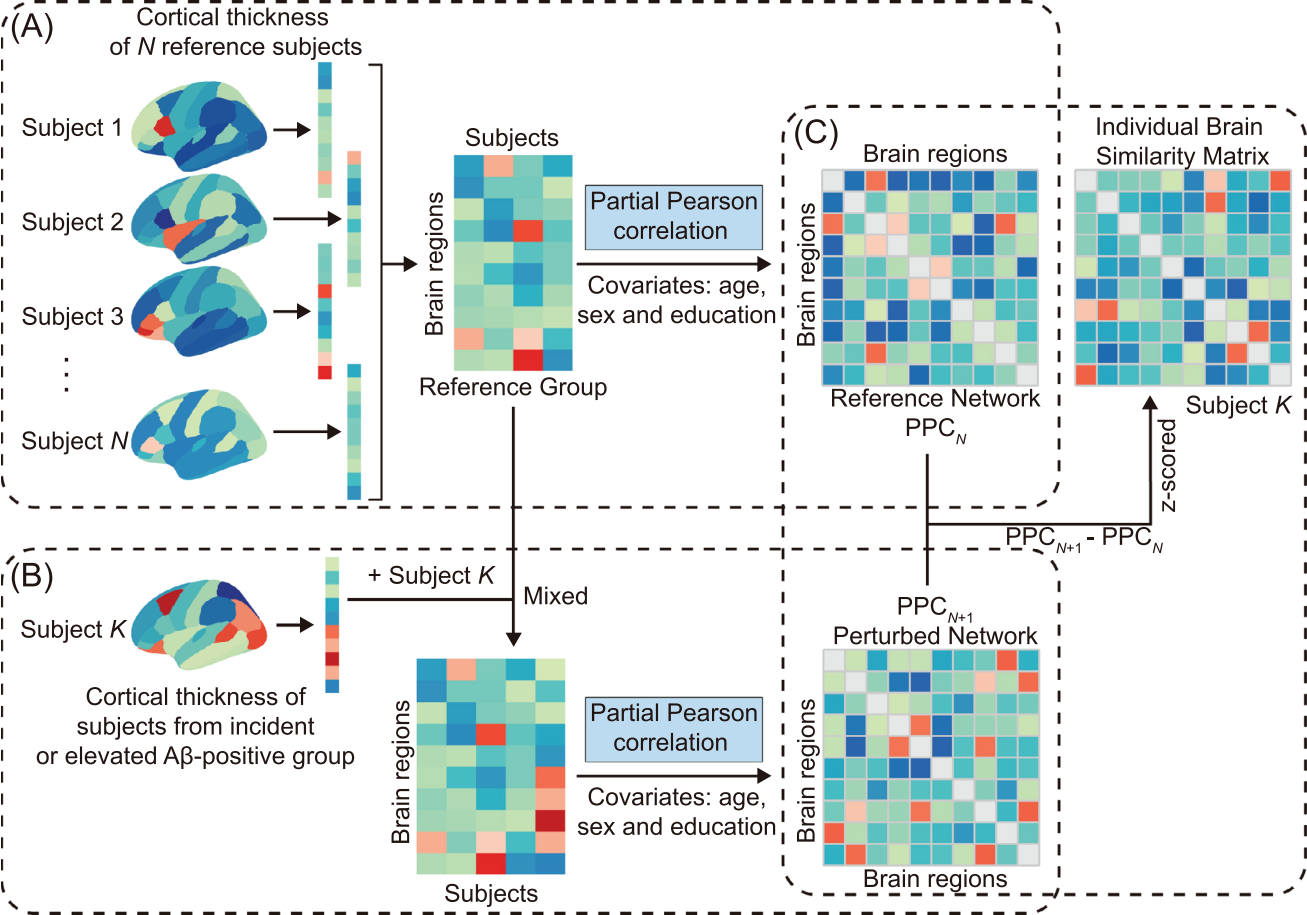
The workflow for constructing individual brain similarity matrices. Regional cortical thickness measures were first extracted for the reference group and individual subjects. A, A reference structural covariance network was constructed across the reference group, with each edge representing the partial Pearson correlation coefficient between cortical thickness values across regions, adjusting for age, sex, and education. B, For each subject, a perturbed structural covariance network was constructed by adding the subject's cortical thickness values to the reference group data and recalculating the network using the same partial Pearson correlation procedure. C, The individual brain similarity metric was defined as the *z* score of the difference between the perturbed network and the reference network. Aβ, amyloid beta; PPC, partial Pearson correlation coefficient matrix.

Individual brain similarity matrices were generated based on cortical thickness data obtained from MRI.^17^, ^45^, ^46^ This method builds upon the perturbation framework, which has been shown to capture subject‐level deviations in structural covariance effectively.^45^ This approach is illustrated in Figure 1. All analyses were implemented using the open‐source Braph2.0 toolbox, which provides a standardized framework for constructing individual brain similarity networks.^47^, ^48^

First, we built a reference structural covariance matrix in the ADNI cohort using cognitively normal participants who were Aβ negative at baseline and longitudinally (*n* = 114). This group‐level matrix was generated by estimating partial Pearson correlation coefficients between the cortical thickness of brain region pairs while controlling for age, sex, and education.^21^ It served as a normative structural network, representing typical patterns of inter‐regional organization in healthy aging.

For each cognitively normal participant outside the reference group who was Aβ positive at baseline or longitudinally, we applied a perturbation‐based approach. Specifically, the participant's cortical thickness values were added to the reference data, and a perturbed covariance network was generated by recomputing the partial Pearson correlations, following the same procedure and covariate adjustments as in reference network construction.^21^ The individual brain similarity network was then calculated as the difference between the perturbed and reference networks, yielding a subject‐specific deviation profile. Each edge in this network represents a structural relationship between brain regions, reflecting how the individual's morphology deviates from the normative pattern. To quantify this deviation, we computed *z* scores across the entire network.

The same procedure was independently applied to the OASIS cohort, using a reference group of 243 cognitively normal, amyloid‐negative participants.

To evaluate whether the size of the reference group influenced the stability of the structural covariance matrix, we performed a resampling analysis in both ADNI and OASIS. For each dataset, we randomly sampled subsets of cognitively normal, Aβ‐negative participants across a range of sample sizes (ADNI: 10–110 individuals; OASIS: 10–230 individuals) and reconstructed covariance matrices 1000 times for each sample size using the same partial‐correlation procedure as in the main analyses. Each resampled covariance matrix was then compared to the full reference covariance matrix, defined as the covariance matrix computed using the complete reference group (ADNI: *n* = 114; OASIS: *n* = 243). Matrix stability was quantified using the intraclass correlation coefficient (ICC), which measures consistency on a scale from 0 to 1. For each sample size, ICC values were averaged across the 1000 replications, and mean ICC values > 0.75 were taken to indicate good reliability.^49^

### 2.5 Cytoarchitecture correspondence analysis

Previous studies have shown that variations in laminar thickness and cortical hierarchy are closely linked to structural covariance, which may reflect fundamental cytoarchitectural patterns in the human cortex.^50^, ^51^ To evaluate this hypothesis, we reclassified brain regions from the Desikan atlas into seven cytoarchitectural classes based on the framework established by von Economo and Koskinas.^52^ To explore the relationship between brain similarity and cortical cytoarchitecture, we mapped our study's main results (brain regions showing significant relations between cognition and brain similarity) onto predefined cytoarchitectural classes. Specifically, we quantified inter‐regional similarity proportion by summarizing the distribution of significant results across all pairs of cytoarchitectural regions, reflecting the network‐level organization of cognitive–brain similarity associations. We also computed the regional contribution by counting the number of significant results involving each region, indicating the extent of regional involvement in cognition‐related similarity patterns.

### 2.6 Statistical analysis

Within each cohort (ADNI, OASIS), we compared the incident and elevated Aβ‐positive groups to the Aβ‐negative group in demographics, cognitive tests, and imaging and fluid biomarkers. The Mann–Whitney *U* test was used for continuous variables, while the chi‐squared test was applied to categorical variables.

To assess whether brain similarity was associated with cognitive changes across different stages of Aβ pathology, analyses were performed separately within the incident and elevated Aβ‐positive groups. For each group and cognitive domain, we fitted separate linear mixed‐effects models in which longitudinal brain similarity values served as the primary predictor. Each brain similarity value or edge within the similarity network was modeled independently. This procedure was repeated for each cognitive domain or test, including attention, memory, visuospatial ability, executive function, language, ADL, and the PACC. All models included age, sex, and education as covariates, as well as an interaction term between the predictor and time. A random intercept was specified for each participant to account for within‐subject variability over time.

Similar analyses were conducted using longitudinal measures of cortical thickness, hippocampal volume, ventricular volume, and total gray matter volume as predictors. For the volumetric models, total intracranial volume was included as an additional covariate. Additionally, we assessed the predictive value of baseline CSF and plasma biomarkers for cognitive trajectories over time because longitudinal biomarkers levels were not available. Linear mixed‐effects models for these analyses included interaction terms between baseline biomarker levels and time to evaluate whether baseline biomarker levels predicted longitudinal cognitive change.

For each brain similarity model, we extracted the *p* values associated with the interaction term between time and brain similarity. A variable was considered significant only if this interaction term survived false discovery rate (FDR) correction using the Benjamini–Hochberg procedure,^53^ applied across all brain similarity values or edges. Separate FDR corrections were performed for models assessing cortical thickness, volumetric measures, and CSF/plasma biomarkers.

To compare the brain similarity models to the thickness, volume, CSF, and plasma biomarker models in predicting cognitive decline, we implemented a nested model comparison framework. For cortical thickness, for example, we first identified all significant brain similarity and cortical thickness variables associated with each cognitive domain. These variables were then compared pairwise. For each pair, we constructed three linear mixed‐effects models: one including only the brain similarity value, one including only the cortical thickness variable, and a full model including both predictors and their interactions with time. Likelihood ratio tests (LRTs) were used to compare the full model against each nested single‐variable model. *P* values from the LRTs were corrected using FDR. We then counted the number of pairwise comparisons in which one variable significantly improved model fit over the other. To evaluate whether brain similarity provided significantly greater predictive value than cortical thickness, we compared the frequency of these instances using a chi‐squared test, or Fisher exact test when expected counts were below five. The same procedure was used to compare brain similarity to volumetric measures and fluid biomarkers. We also extracted the median coefficient of determination (*R* ^2^) for each marker to quantify their overall explanatory power. For chi‐squared tests, we reported the *χ* ^2^ statistic and corresponding *p* value; for Fisher exact tests, we reported the odds ratio (OR) and *p* value. Together, these metrics were used to visualize and compare the predictive performance of brain similarity relative to the other markers (cortical thickness, hippocampal volume, ventricular volume, total gray matter volume, CSF p‐tau181, CSF t‐tau, plasma p‐tau181, plasma NfL). For the models that included fluid biomarker measurements, given that they were only available at baseline, we restricted comparisons to baseline brain similarity values. Specifically, we selected brain similarity features that showed significant longitudinal associations and extracted their baseline values to ensure temporally aligned comparisons between variables.

Finally, we evaluated the predictive performance of brain similarity, cortical thickness, brain volumes, and fluid biomarkers for predicting diagnostic conversion, such as progression from cognitively normal to MCI or AD. For each marker type, we first applied a least absolute shrinkage and selection operator (LASSO)‐based dimensionality reduction procedure to select informative features.^54^ The regularization strength (λ), which controls the degree of penalization applied to model coefficients and hence the number of features retained, was optimized using 5‐fold cross‐validation. These selected features were then entered into logistic regression classifiers to distinguish converters from non‐converters. To obtain robust estimates of model performance and account for sampling variability, we implemented a bootstrap procedure with 1000 iterations. In each iteration, 80% of the sample was randomly selected for training and the remaining 20% for testing, and a logistic regression model was fitted. Predicted probabilities from each bootstrap iteration were used to generate receiver operating characteristic (ROC) curves. The median ROC curve across the 1000 iterations was computed to represent the central tendency of model performance, while the 95% confidence interval was derived from the distribution of ROC curves. Correspondingly, the area under the curve (AUC) and its 95% confidence interval were calculated to compare classification accuracy across models.

## 3 RESULTS

Baseline characteristics of participants from the ADNI and OASIS cohorts are summarized in Table 1. ADNI included 114 Aβ‐negative stable, 26 incident Aβ‐positive, and 45 elevated Aβ‐positive individuals, while OASIS included 243, 36, and 71 individuals in the respective groups. Importantly, in both ADNI and OASIS, the incident Aβ‐positive groups did not differ from the stable Aβ‐negative groups on any cognitive measure, nor in regional cortical thickness or volumetric measures at baseline. Significant group differences were observed only in the elevated Aβ‐positive group. In the ADNI cohort, the elevated Aβ‐positive group showed significant lower PACC scores (*p* = 0.004) and higher ADASQ4 (*p* = 0.007), TMT‐A (*p* = 0.002), and TMT‐B (*p* = 0.03) scores compared to the Aβ‐negative stable group, indicating lower cognitive performance. Despite these cognitive differences, the elevated Aβ‐positive group showed no differences in regional cortical thickness or volumetric measures at baseline, consistent with the pattern observed in the incident Aβ‐positive group. Baseline biomarker levels showed an increase across Aβ groups. In the incident Aβ‐positive group, CSF p‐tau181 (*p* = 0.018), plasma p‐tau181 (*p* = 0.006), and plasma t‐tau (*p* = 0.007) were significantly elevated compared to the stable Aβ‐negative group. In the elevated Aβ‐positive group, all biomarkers showed stronger increases, including plasma NfL (*p* < 0.001), plasma p‐tau181 (*p* < 0.001), CSF p‐tau181 (*p* < 0.001), and t‐tau (*p* < 0.001) consistent with more severe pathological changes.

We further evaluated the stability of the reference groups used to construct the covariance matrices. For each dataset, we determined the smallest sample size at which the mean ICC across the 1000 resampled covariance matrices exceeded the reliability threshold of 0.75.^49^ As shown in Figure S2 in supporting information, this threshold was reached at sample sizes > 34 individuals in ADNI and 54 individuals in OASIS. Because the reference groups used in our analyses (ADNI: *n* = 114; OASIS: *n* = 243) are substantially larger than these stability points, the resulting covariance matrices provide robust and reliable normative baselines for subsequent individual‐level similarity estimation.

### 3.1 Brain similarity predicts cognitive decline better than regional cortical thickness and volumes

In the ADNI cohort, almost all the models that included brain imaging measures significantly predicted cognitive decline across the Aβ‐positive groups, albeit to different extents (please see Table S2 in supporting information for specific *R* ^2^ and *p* values for each model).

**FIGURE 2:**
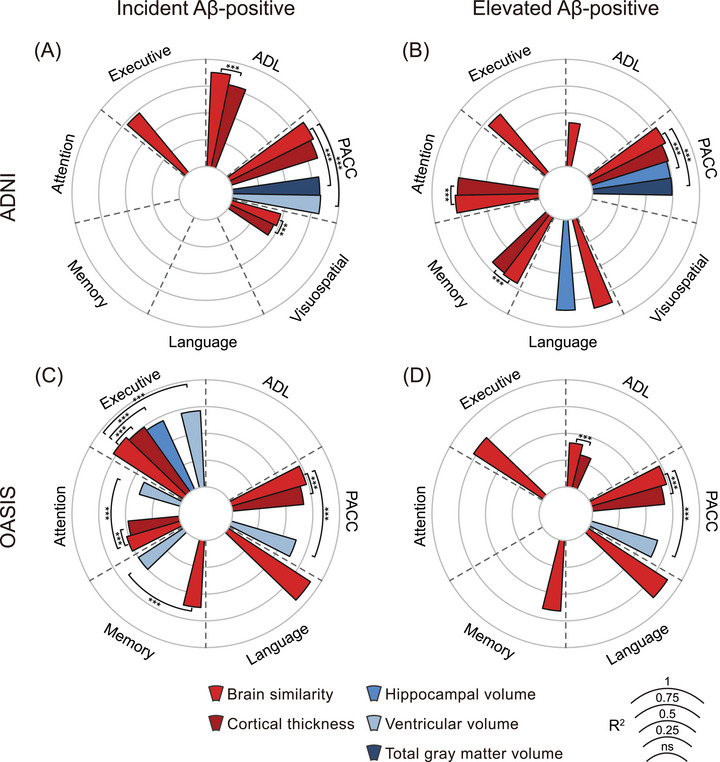
Comparison of brain similarity, regional cortical thickness, and brain volume measures in predicting cognitive decline across different amyloid groups. The figure presents the *R* ^2^ values from LME models based on brain similarity and regional thickness features, indicating the proportion of variance explained in different cognitive domains, including executive function, attention, memory, language, visuospatial abilities, ADL, and PACC. Results from the ADNI cohort are shown for the incident (A) and elevated (B) Aβ‐positive groups. Corresponding analyses from the OASIS cohort are presented in (C) and (D), respectively. The radar plots illustrate the predictive performance of models based on brain similarity, regional cortical thickness, and brain volume measures (total gray matter, ventricle, and hippocampus), enabling a direct comparison of their respective contributions to cognitive decline prediction. Significance levels: *p* < 0.05 (*), *p* < 0.01 (**), *p* < 0.001 (***). A, amyloid; Aβ, amyloid beta; ADL, activities of daily living; ADNI, Alzheimer's Disease Neuroimaging Initiative; LME, linear mixed effects; OASIS, Open Access Series of Imaging Studies; PACC, Preclinical Alzheimer Cognitive Composite; *R* ^2^, coefficient of determination.

Briefly, in the incident Aβ‐positive group, brain similarity showed strong associations with executive function, visuospatial ability, PACC, and ADL, whereas cortical thickness was significantly associated with visuospatial ability, PACC, and ADL, and finally ventricular volume and total gray matter volume showed significant associations with PACC (please see Table S2 for specific R^2^ and *p* values for each model). However, when the predictive ability between these different models was compared, brain similarity consistently outperformed the other imaging models: it was a better predictor of visuospatial ability (*χ* ^2^ = 141.85, *p* < 0.001), ADL (*χ* ^2^ = 895.45, *p* < 0.001), and PACC (*χ* ^2^ = 165.95, *p* < 0.001) compared to cortical thickness and it also explained more variance in PACC compared to both total gray matter volume (*χ* ^2^ = 1026.03, *P *< 0.001) and ventricular volume (*χ* ^2^ = 777.69, *P *< 0.001; Figure 2A).

In the elevated Aβ‐positive group, the results were similar with brain imaging measures significantly predicting decline in several cognitive measures. Specifically, brain similarity was a significant predictor of changes in executive function, language, attention, memory, PACC, and ADL, whereas cortical thickness predicted longitudinal attention, memory, and PACC scores, hippocampal volume predicted language and PACC, and total gray matter volume was only significantly associated with PACC (please see Table S2 for specific *R* ^2^ and *p* values for each model). When we compared the different models, brain similarity was again a better predictor of attention (*χ* ^2^ = 167.12, *p* < 0.001) and memory (*χ* ^2^ = 237.16, *p* < 0.001) decline compared to cortical thickness, and outperformed hippocampal volume (*χ* ^2^ = 812.23, *p* < 0.001) and total gray matter volume (*χ* ^2^ = 821.02, *p* < 0.001) in the predictions of PACC (Figure 2B).

Results from the incident and elevated Aβ‐positive groups of OASIS generally confirmed findings from ADNI. In the OASIS dataset, brain similarity, cortical thickness, and volumetric measures each demonstrated predictive value for cognitive decline across specific domains in both Aβ‐positive groups (please see Table S3 in supporting information for specific *R* ^2^ and *p* values for each model). In both groups, brain similarity consistently outperformed regional cortical thickness and volumetric measures. Specifically, in the incident Aβ‐positive group, brain similarity explained more variance in executive function (*χ* ^2^ = 234.01, *p* < 0.001), attention (*χ* ^2^ = 213.45, *p* < 0.001), and PACC (*χ* ^2^ = 229.51, *p* < 0.001) compared to cortical thickness. It also demonstrated superior predictive value for executive function (*χ* ^2^ = 88.32, *p* < 0.001), attention (*χ* ^2^ = 88.32, *p* < 0.001), memory (*χ* ^2^ = 88.32, *p* < 0.001), and PACC (*χ* ^2^ = 14.51, *p* < 0.001) relative to ventricular volume, and outperformed hippocampal volume in predicting executive function (*χ* ^2^ = 88.32, *p* < 0.001; Figure 2C). In the elevated Aβ‐positive group, brain similarity showed stronger associations with ADL (*χ* ^2^ = 352.29, *p* < 0.001) and PACC (*χ* ^2^ = 5054.78, *p* < 0.001) than cortical thickness, and explained more variance in PACC (*χ* ^2^ = 66, *p* < 0.001) than ventricular volume (Figure 2D).

Across both datasets, we observed highly convergent results in both the incident and elevated Aβ‐positive groups. In the incident group, brain similarity consistently predicted decline in executive function and PACC in both ADNI and OASIS, outperforming regional cortical thickness and volumetric markers. Similarly, in the elevated Aβ‐positive group, brain similarity showed reproducible associations with longitudinal changes in executive function, memory, language, ADL, and PACC across datasets, again exceeding the predictive value of conventional MRI measures. These convergent findings across two independent cohorts and across amyloid stages highlight the robustness of brain similarity as a sensitive imaging marker of early cognitive decline in preclinical AD.

To evaluate whether brain similarity measures can still predict longitudinal cognitive change in cognitively normal individuals at increased genetic risk but without amyloid pathology, we examined brain similarity in a cognitively normal, Aβ‐negative cohort from PPMI. In this sample, baseline brain similarity significantly predicted longitudinal change in global cognition (*R* ^2^ = 0.61) and executive function (*R* ^2^ = 0.76), whereas baseline cortical thickness did not predict change in any cognitive domain. These findings indicate that brain similarity captures subtle individual differences even in normative populations and support the broader applicability of the measure.

### 3.2 Brain similarity predicts cognitive decline better than CSF and plasma biomarkers

**FIGURE 3:**
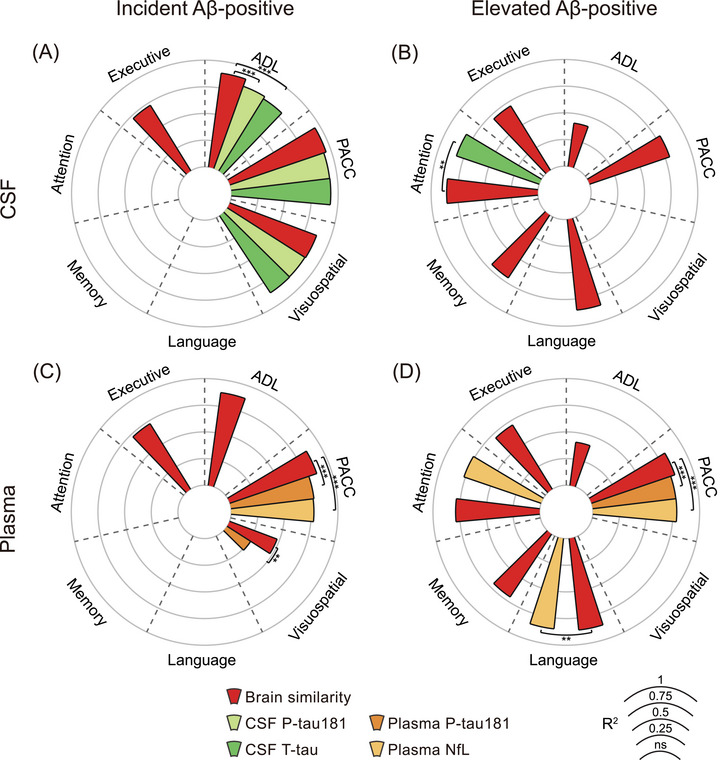
Comparison of brain similarity and CSF/plasma biomarkers in predicting cognitive decline across different amyloid groups. The figure presents the *R* ^2^ values from LME models based on brain similarity and regional thickness features, indicating the proportion of variance explained in different cognitive domains, including executive function, attention, memory, language, visuospatial abilities, ADL, and PACC. Results from the ADNI cohort are shown for the incident (A) and elevated (B) Aβ‐positive groups. Corresponding results from the OASIS cohort are presented in (C) and (D), respectively. The radar plots illustrate the predictive performance of models based on brain similarity and biomarkers (CSF/plasma p‐tau181, CSF t‐tau, and plasma NfL), enabling a direct comparison of their respective contributions to cognitive decline prediction. Significance levels: *p* < 0.05 (*), *p* < 0.01 (**), *p* < 0.001 (***). A, amyloid; Aβ, amyloid beta; ADL, activities of daily living; ADNI, Alzheimer's Disease Neuroimaging Initiative; CSF, cerebrospinal fluid; LME, linear mixed effects; NfL, neurofilament light chain; OASIS, Open Access Series of Imaging Studies; PACC, Preclinical Alzheimer Cognitive Composite; p‐tau, tau hyperphosphorylation; *R* ^2^, coefficient of determination; t‐tau, total tau.

In the subset of participants with available fluid biomarker data (at baseline), we found that baseline brain similarity significantly predicted executive function, visuospatial ability, PACC, and ADL, whereas both baseline CSF p‐tau181 and CSF t‐tau significantly predicted visuospatial ability, PACC, and ADL (please see Table S4 in supporting information for specific *R* ^2^ and *p* values for each model) in the incident Aβ‐positive group. By comparison, brain similarity was a stronger predictor of ADL than both CSF p‐tau181 (*χ* ^2^ = 66.53, *p* < 0.001) and CSF t‐tau (χ^2^ = 86.65, *p* < 0.001; Figure 3A). Similarly, in the elevated Aβ‐positive group, brain similarity was significantly associated with executive function, attention, memory, language, PACC, and ADL. However, CSF t‐tau only showed a significant association with attention decline and CSF p‐tau181 did not show significant associations with any domains or tests. When we compared the ability of brain similarity to predict attention decline, we found that brain similarity was a superior predictor (OR = 0.02, *p* = 0.005) to CSF p‐tau (Figure 3B).

Regarding plasma biomarkers, although brain similarity, plasma p‐tau181, and plasma NfL predicted longitudinal changes in different cognitive domains in the incident Aβ‐positive group (please see Table S4 for specific *R* ^2^ and *p* values for each model), brain similarity exhibited stronger associations with visuospatial ability (OR = 0.06, *p* = 0.008) and PACC (*χ* ^2^ = 924.28, *P *< 0.001) compared to plasma p‐tau181, and was more strongly associated with PACC than plasma NfL (*χ* ^2^ = 1163.16, *P *< 0.001; Figure 3C). In the elevated Aβ‐positive group, brain similarity remained the strongest predictor across cognitive domains, showing significantly stronger associations with PACC compared to both plasma p‐tau181 (*χ* ^2^ = 18.42, *p* < 0.001) and NfL (*χ* ^2^ = 36.29, *p* < 0.001), and also showed a stronger relationship with language relative to plasma NfL (OR = 0.02, *P *= 0.005; Figure 3D).

### 3.3 Brain similarity predicts conversion to MCI or AD

**FIGURE 4:**
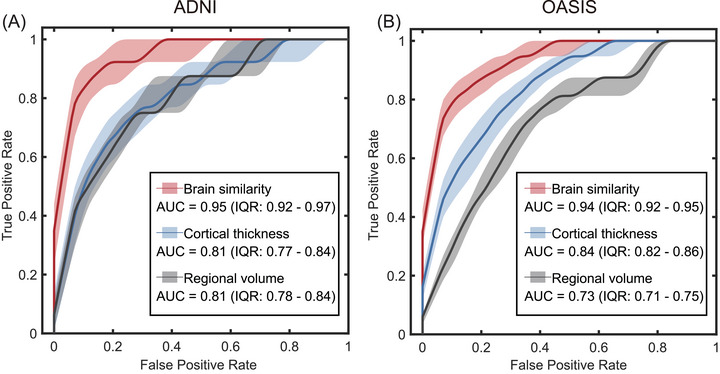
Comparison of brain similarity, regional thickness, and volume measures in prediction of conversion to MCI and AD. ROC curves for classification models predicting conversion to MCI and AD in the (A) ADNI and (B) OASIS3 cohorts. The plots compare the performance of brain similarity, cortical thickness, and volumetric features with corresponding AUC values and interquartile ranges provided in the legends. Shaded regions indicate the 95% variability across bootstrapped samples. AD, Alzheimer's disease; ADNI, Alzheimer's Disease Neuroimaging Initiative; AUC, area under the curve; IQR, interquartile range; MCI, mild cognitive impairment; OASIS, Open Access Series of Imaging Studies; ROC, receiver operating characteristic.

To assess the capacity of brain similarity measures to identify individuals at risk of progressing to MCI or AD, a predictive modeling framework was used. In the ADNI dataset, brain similarity demonstrated high predictive accuracy for diagnostic conversion (AUC = 0.95, interquartile range [IQR]: 0.92–0.97), outperforming cortical thickness (AUC = 0.81, IQR: 0.77–0.84) and volume‐based models (AUC = 0.81, IQR: 0.78–0.84; Figure 4A). A similar pattern was observed in the OASIS3 dataset, in which brain similarity yielded a better predictive performance (AUC = 0.94, IQR: 0.92–0.95), compared to cortical thickness (AUC = 0.84, IQR: 0.82–0.86) and volumetric features (AUC = 0.73, IQR: 0.71–0.75; Figure 4B).

We further compared brain similarity to CSF/plasma biomarker‐based predictions. Given the limited sample size (*n* = 60), these findings should be interpreted as exploratory. The brain similarity model yielded a predictive performance (AUC = 0.76, IQR: 0.73–0.79), while the model based on all fluid biomarkers performed slightly better (AUC = 0.81, IQR: 0.79–0.82). Notably, when brain similarity features were combined with fluid biomarkers in a single model, predictive accuracy improved substantially (AUC = 0.88, IQR: 0.84–0.91), suggesting that brain similarity provides complementary structural information beyond what is captured by biomarkers alone.

### 3.4 Brain similarity results are mainly located within cortical layer II areas in early AD

**FIGURE 5:**
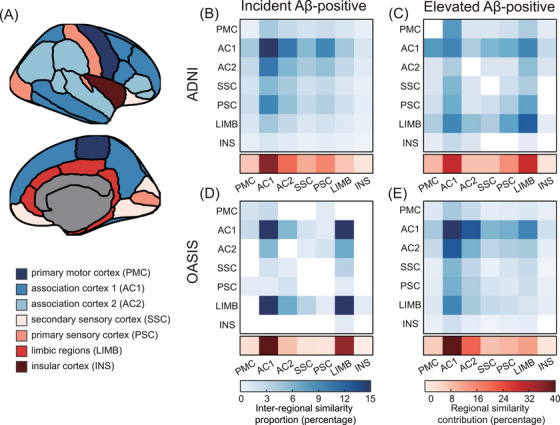
Distribution of brain similarity across cytoarchitectural regions. A, The cortex is parcellated into seven cytoarchitectural regions based on shared laminar properties: primary motor cortex (PMC), association cortex 1 (AC1), association cortex 2 (AC2), secondary sensory cortex (SSC), primary sensory cortex (PSC), limbic regions (LIMB), and insular cortex (INS). B and C, Results from the ADNI cohort, with the incident Aβ‐positive group in (B) and the elevated Aβ‐positive group in (C). D and E, Results from the OASIS cohort, with the incident Aβ‐positive group in (D) and the elevated Aβ‐positive group in (E). Heatmaps illustrate the distribution of significant brain similarity features across cytoarchitectural regions. The blue matrices represent the proportion of features spanning each pair of regions (inter‐regional similarity proportion), while the red horizontal strips below each matrix summarize the overall involvement of each region (regional similarity contribution). A, amyloid; Aβ, amyloid beta; ADNI, Alzheimer's Disease Neuroimaging Initiative; OASIS, Open Access Series of Imaging Studies.

As shown in Figure 5A, the brain was parcellated into seven cytoarchitectural regions, including primary motor cortex (PMC), primary sensory cortex (PSC), association cortex 1 (AC1), association cortex 2 (AC2), secondary sensory cortex (SSC), limbic regions (LIMB), and the insular cortex (INS). These regions reflect distinct cortical layers and functional profiles. Figures 5B–E depict the distribution of significant brain similarity variables across these regions, summarizing both network‐level distribution and region‐level involvement.

In the ADNI dataset, significant brain similarity variables in the incident Aβ‐positive group were most frequently localized to AC1 (36.6%), followed by AC2 (20.0%) and PSC (15.6%). Lesser involvement was observed in SSC (10.7%), LIMB (7.4%), PMC (7.3%), and INS (2.4%; Figure 5B). In the elevated Aβ‐positive group, the highest proportions were observed in AC1 (29.2%) and LIMB (28.9%), followed by PSC (14.4%), PMC (9.2%), SSC (7.7%), AC2 (7.7%), and INS (2.8%; Figure 5C).

In the OASIS dataset, a comparable distribution pattern emerged. For the incident Aβ‐positive group, the most prominent contributions were from AC1 (47.3%) and LIMB (35.4%), with lesser involvement from AC2 (8.0%), PMC (3.5%), SSC (3.1%), PSC (2.2%), and INS (0.4%; Figure 5D). In the elevated Aβ‐positive group, AC1 (41.0%), AC2 (21.0%), and LIMB (12.2%) remained the most involved regions, followed by PSC (9.3%), SSC (7.8%), PMC (6.4%), and INS (2.3%; Figure 5E).

Overall, significant brain similarity features were primarily localized to AC1, a set of association cortical regions associated with layer II, which supports intracortical connectivity and higher order processing.^55^

## 4 DISCUSSION

Current anti‐amyloid therapies have shown moderate effectiveness in slowing cognitive decline among individuals with MCI or dementia due to AD. Moreover, these treatments carry risks of serious adverse effects, including cerebral edema and effusions.^56^, ^57^ Given these limitations, research has recently shifted toward the preclinical stage of AD, during which interventions may yield greater therapeutic benefits before extensive neurodegeneration occurs.^58^ However, a significant challenge in clinical trial design remains the lack of sensitive, cost‐effective, and widely accessible outcome measures capable of detecting subtle disease‐related changes at this early stage. Conventional biomarkers, such as MRI‐derived atrophy metrics and biofluid markers, exhibit limitations in preclinical populations,^59^, ^60^ reducing their reliability for early detection. Thus, there is a critical need for novel biomarkers that can detect neuroanatomical changes before significant atrophy occurs. To address this gap, we propose brain similarity as a novel imaging measure that captures morphometric relationships between brain regions. In our study, brain similarity outperformed conventional biomarkers in predicting cognitive decline and conversion to MCI or AD. These findings suggest that brain similarity is a marker of subtle neuroanatomical changes in the early stages of AD that may be used to track cognitive changes in clinical trials.

Amyloid pathology is a hallmark of AD, characterized by the accumulation of Aβ plaques in the brain.^2^, ^3^ These plaques form due to the abnormal aggregation of Aβ peptides, which result from the cleavage of amyloid precursor protein.^61^ While amyloid accumulation is one of the earliest detectable pathological features of AD, its precise role in driving neurodegeneration remains unclear.^3^ Evidence suggests that excessive Aβ disrupts synaptic function, alters neuronal communication, and triggers neuroinflammatory responses, contributing to cognitive decline and disease progression.^62^ However, conventional volumetric MRI measures primarily detect later stage atrophy and may not capture early structural effects of amyloid accumulation.^63^ In contrast, covariance patterns can reveal system‐level changes in brain architecture, even in the absence of overt atrophy.^64^, ^65^, ^66^ Our findings align with this, demonstrating that brain similarity captures early neuroanatomical changes more robustly than conventional thickness and volume measures. Notably, brain similarity showed consistent predictive ability across amyloid groups, suggesting that it may serve as a sensitive biomarker for detecting subtle structural changes associated with early AD progression. These properties were supported by our supplementary analysis in the PPMI cohort, in which brain similarity predicted longitudinal cognitive decline in *APOE* ε4 carriers despite the absence of amyloid pathology or measurable atrophy.

Brain similarity metrics offer a system‐level perspective on cortical organization by capturing morphometric relationships between regions rather than isolated thickness or volume measures. Their biological validity is supported by converging evidence that structural similarity reflects key microstructural properties of the cortex.^16^ Prior studies using frameworks such as morphometric similarity networks (MSNs)^18^ and Morphometric Inverse Divergence (MIND) demonstrate that multifeatured or vertex‐level morphometric similarity aligns closely with cortical lamination patterns, depth‐dependent myelination gradients, and gene transcriptional profiles.^67^, ^68^ Related approaches, such as the Regional Vulnerability Index (RVI), further demonstrate that individualized structural profiles can capture the expression of disease‐related atrophy patterns.^69^, ^70^ Together, these findings suggest that structural similarity may reflect not only microstructural similarity but also the large‐scale connectivity patterns between brain areas, although the two things are not equivalent.^50^, ^71^ Building on this foundation, our findings show that brain similarity consistently provides more accurate prediction than traditional atrophy‐based measures in predicting cognitive function, particularly in elevated Aβ‐positive individuals. By integrating morphometric information across distributed regions, brain similarity detects system‐level structural disruptions that conventional metrics may not capture. This approach is sensitive to early‐stage microstructural alterations and coordinated neurodegenerative processes, including selective vulnerability of specific cortical layers, that otherwise require *post mortem* histology or ultra‐high‐field MRI to be detected. These strengths underscore its potential as a system‐level marker of emerging neurodegeneration in preclinical AD. These advantages were not limited to AD‐related cohorts, but also in an independent sample from PPMI, confirming their generalizability.

Our study demonstrates that brain similarity is a sensitive marker of cognitive decline in preclinical AD, with highly convergent findings across the ADNI and OASIS cohorts. In the incident Aβ‐positive group, brain similarity showed stronger associations with cognitive trajectories than regional cortical thickness and volumetric measures, with convergent effects across ADNI and OASIS for PACC and executive function. These findings support prior reports suggesting morphometric similarity captures system‐level alterations before gross atrophy emerges.^15^, ^66^ In the elevated Aβ‐positive group, brain similarity remained the strongest predictor across most cognitive domains, with consistent associations across both cohorts for PACC, ADL, executive function, and memory, even when conventional markers failed to reach significance. This consistency across different amyloid stages suggests that brain similarity can detect early neuroanatomical changes irrespective of the timing of Aβ accumulation. Importantly, our findings extend to fluid biomarkers. Across the comparisons to CSF and plasma biomarkers, brain similarity showed stronger and more consistent associations than p‐tau181, t‐tau, and NfL in predicting cognitive decline. While some biomarkers were associated with PACC, they generally failed to predict domain‐specific changes such as executive decline or changes in daily living activities, where brain similarity remained informative. These results align with recent studies highlighting the spatial and biological specificity of structural similarity networks^66^, ^67^ and suggest that brain similarity may be particularly suitable for capturing distributed, cortical‐level pathology not reflected in fluid biomarkers.^18^ Finally, in both ADNI and OASIS cohorts, brain similarity achieved higher AUCs than conventional imaging markers in predicting conversion to MCI and AD. The additional evidence from the PPMI cohort further demonstrates that brain similarity predicts cognitive decline even in the absence of amyloid pathology. These consistent findings across independent datasets, measures, and disease stages highlight the robustness and generalizability of the brain similarity network. Collectively, these results support the integration of brain similarity as a sensitive, biologically grounded outcome marker in future preclinical AD trials.

Despite the strengths of our study, several limitations should be acknowledged. First, our sample sizes were modest, primarily due to the stringent inclusion criteria applied to simulate the characteristics of preclinical AD trial populations.^72^ While this allowed us to focus on individuals most representative of early‐stage intervention cohorts, it may have limited our statistical power. Second, longitudinal data for CSF and plasma biomarkers were unavailable for all participants, so we could not compare longitudinal biomarker changes to longitudinal measures of brain similarity. In addition, several imaging biomarkers relevant to preclinical AD, such as entorhinal tau PET, could not be included due to a mismatch between imaging acquisition dates and the timing of cognitive assessments. Nevertheless, the stronger associations observed between brain similarity and cognitive outcomes, together with convergent results from longitudinal cortical thickness and volumetric trajectories, support the stability of our findings. Moreover, our findings are also limited to individuals who were cognitively normal at baseline, as our analyses focused specifically on the preclinical stage of AD. In later disease stages such as MCI or AD, during which substantial atrophy has already occurred, affecting regional cortical thickness and volumetric measures, the value of brain similarity remains to be established but is likely to be less prominent than in early disease stages when there is minimal atrophy. Finally, our framework relied on cortical thickness to derive similarity measures. We focused on thickness rather than gray matter volume because it has a clearer biological interpretability: it reflects the distance between the gray matter/white mattery boundary and the pial surface, whereas gray matter volume reflects a combination of surface area, thickness, and volume.^73^ Thickness is also sensitive to subtle laminar alterations that characterize preclinical AD.^74^, ^75^ Future work may extend our approach by integrating additional morphometric features or multimodal MRI‐based methods, including MIND and related frameworks,^67^, ^68^, ^69^ to further enhance the sensitivity and clinical utility of similarity‐based biomarkers in preclinical AD.

As the focus of AD research increasingly shifts toward prevention and early intervention, there is a pressing need to develop and implement outcome measures that are sensitive to the subtle neurobiological alterations that characterize the preclinical stages of the disease. Conventional biomarkers, while effective in later disease stages, often fail to capture early, system‐level alterations before overt neurodegeneration becomes apparent. Our findings highlight the potential of brain similarity metrics to fill this gap, offering a biologically meaningful, imaging‐derived measure that outperforms traditional atrophy‐based markers in predicting cognitive decline. Moving forward, incorporating such alternative biomarkers into clinical trial designs may enhance the detection of treatment effects during the earliest stages of AD, when intervention holds significant promise for altering disease trajectories.

## CONFLICT OF INTEREST STATEMENT

There are no conflicts to report for this work by the authors.

## CONSENT STATEMENT

All participants gave their informed consent, and the study protocol was approved by the committee on human research at each participating institution.

## Supporting information



Supporting Information

Supporting Information
