# Supplementary material for: Tracking early cognitive decline in preclinical AD with brain MRI similarity
Source: Alzheimers Dement. 2026 Mar 18;22(3):e71170. doi: 10.1002/alz.71170 (PMC13093512; doi:10.1002/alz.71170)
Supplement: Supplementary file 1 — Supporting Information [file ALZ-22-e71170-s001.docx]

**Tracking early cognitive decline in preclinical AD with brain MRI similarity**

Jiawei Sun*, Blanca Zufiria-Gerboles, Massimiliano Passaretti, Giovanni Volpe, Mite Mijalkov, Joana B. Pereira*, for the Alzheimer's Disease Neuroimaging Initiative

* Corresponding authors:

Jiawei Sun, Email: jiawei.sun@ki.se | Tel: +46 765926812

Joana B. Pereira, Email: joana.pereira@ki.se | Tel: +46 709966186

This file includes:

Section S1

Figures S1 and S2

Tables S1 to S4

**Section 1. PPMI normative cohort: participants, methods, and results**

**Participants**

Participants were drawn from the healthy control cohort of the Parkinson’s Progression Markers Initiative (PPMI)[1]. PPMI is a large, longitudinal study focused on biomarker discovery in Parkinson’s disease, and its healthy control cohort consists of rigorously screened individuals without neurological disorders. Healthy controls are required to have no clinically significant neurological disorder, no first-degree relative with Parkinson’s disease, and normal dopamine transporter (DAT) SPECT imaging by visual inspection, thereby representing a stringently screened normative sample.

From this cohort, we selected cognitively normal and Aβ-negative individuals, including 98 APOE4 non-carriers and 27 APOE4 carriers. Baseline participant characteristics are provided in Supplementary Table S1. Cognitive performance was assessed using standardized neuropsychological tests spanning multiple domains. Global cognition was evaluated with the Montreal Cognitive Assessment (MoCA); visuospatial abilities with Benton’s Judgment of Line Orientation (JLO); memory with the Hopkins Verbal Learning Test-Revised (HVLT-R); executive functions with semantic fluency and the Letter-Number Sequencing test (LNS); and attention with the Symbol Digit Modalities Test (SDMT)[2].

**MRI Acquisition and Preprocessing**

Structural MRI scans in PPMI were acquired on 3T MRI scanners using a high-resolution T1-weighted 3D sequence (MP-RAGE or IR-FSPGR), following the standardized PPMI imaging protocol. The acquisition parameters were: slice thickness = 1.0 mm, in-plane resolution = 1.0 × 1.0 mm², matrix size = 256 × 256, field of view (FOV) = 256 mm, and approximately 192 sagittal slices covering the entire brain, including the cerebellum and brainstem structures. All scans were acquired with isotropic 1 mm³ voxels, and participating sites were required to use site-certified scanners and harmonized sequence parameters to ensure cross-site and longitudinal consistency.

T1-weighted images underwent standard preprocessing steps, including bias-field correction, skull stripping, segmentation, and cortical surface reconstruction. Quality control was performed following PPMI guidelines to exclude scans affected by substantial motion or artifacts. Cortical thickness was then estimated using the same pipeline applied to the ADNI and OASIS cohorts to ensure methodological consistency across datasets. Regional cortical thickness values were extracted from the Desikan-Killiany atlas for use in subsequent analyses.

**CSF biomarker measurements**

Amyloid biomarker data in PPMI were obtained from cerebrospinal fluid (CSF), which was collected at baseline following the standardized PPMI biospecimen protocol (https://www.ppmi-info.org). Briefly, 15-20 mL of CSF was withdrawn via lumbar puncture, centrifuged at 2000 × g for 10 minutes at room temperature, and aliquoted into 1.5 mL polypropylene tubes prior to freezing. All samples were shipped to the University of Pennsylvania Biomarker Research Laboratory for analysis. Concentrations of Aβ₄₂ were quantified using Elecsys® electrochemiluminescence immunoassays on the cobas e 601 analyzer (Roche Diagnostics), in accordance with PPMI-standardized operating procedures.

An Aβ_42_ concentration below 683 pg/mL was used as the cutoff for amyloid abnormality[3].

**Construction of individual brain similarity matrices**

Brain similarity for the PPMI APOE4 carrier group was computed using the same perturbation-based framework applied in the ADNI and OASIS cohorts. A reference structural covariance matrix was first constructed from the 98 cognitively normal, Aβ-negative APOE4 non-carriers, representing the normative cortical organization in this cohort. For each APOE4 carrier, cortical thickness values were added to the reference dataset, and a perturbed covariance matrix was recalculated using partial Pearson correlations while adjusting for age, sex, and education. The individual brain similarity matrix was obtained by computing the deviation between the perturbed and reference matrices, yielding a subject-specific pattern of structural covariance differences across all inter-regional connections.

**Association between baseline brain measure and longitudinal cognitive change**

To evaluate whether baseline brain similarity or baseline cortical thickness predicted cognitive decline in the PPMI cohort, we analyzed APOE4 carriers using linear mixed-effects models. For each cognitive domain, baseline brain similarity values were entered as the main predictor, and each network edge was modelled separately. Cognitive outcomes included global cognition, visuospatial ability, memory, executive functions, and attention. All models adjusted for age, sex, and education and included an interaction between the predictor and time, with a subject-specific random intercept to account for within-person variability. A variable was considered significant only if this interaction term survived FDR correction.

**Results**

In the APOE4 carrier group, baseline brain similarity showed significant associations with longitudinal cognitive decline, whereas baseline cortical thickness did not predict change in any domain. Specifically, higher deviation in brain similarity was associated with decline in global cognition (R² = 0.61) and executive functions (R² = 0.76). No significant associations were observed for visuospatial ability, memory, or attention after FDR correction. In contrast, none of the cortical thickness regions were associated with longitudinal change across any cognitive domain. These findings indicate that brain similarity captures subtle inter-individual variability in cognitive trajectories that are not reflected in regional cortical thickness measures.


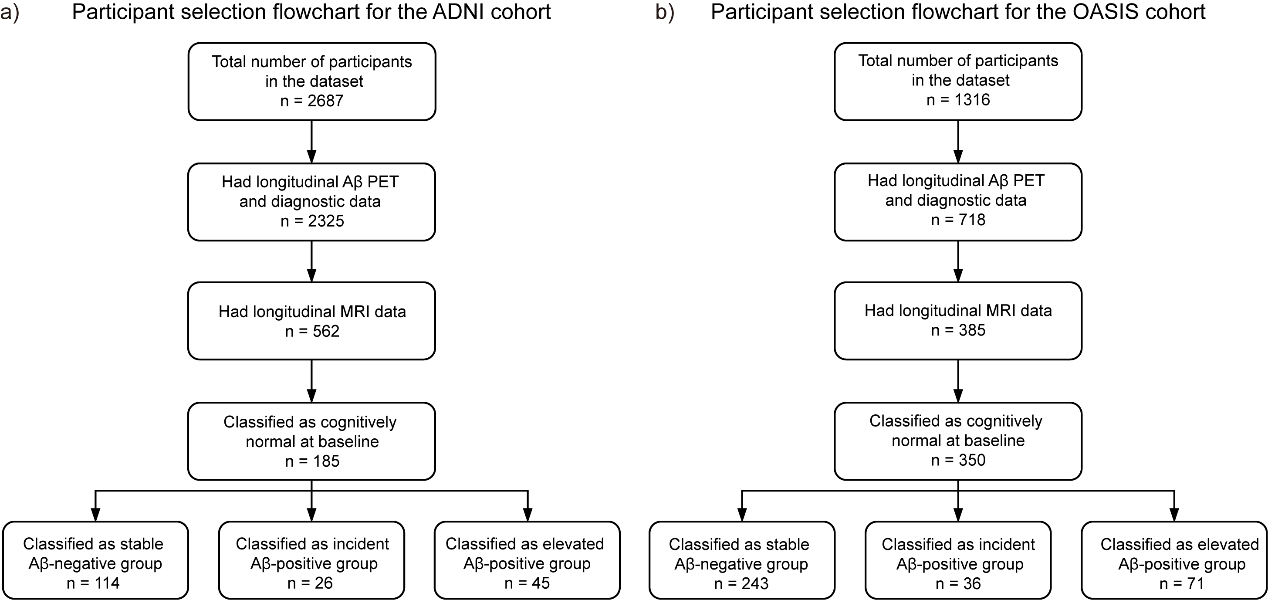


**Figure S1. Participant selection flowcharts for the ADNI and OASIS cohorts.**

The diagrams illustrate the stepwise selection of cognitively normal participants with available amyloid PET and longitudinal data, culminating in the identification of three subgroups based on amyloid status over time: stable Aβ-negative, incident Aβ-positive, and elevated Aβ-positive. Panel (a) shows the selection process in the ADNI cohort, and panel (b) depicts the corresponding process in the OASIS cohort. A, amyloid; ADNI, Alzheimer's Disease Neuroimaging Initiative; OASIS, Open Access Series of Imaging Studies.


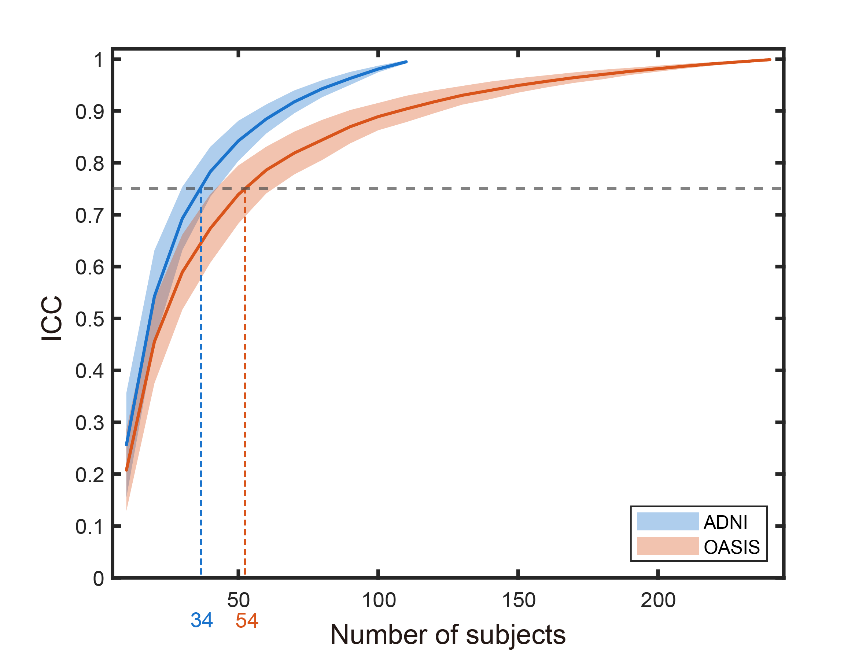


**Figure S2. Stability of the normative structural covariance matrix as a function of sample size.**

ICC were computed across 1,000 bootstrapped covariance matrices for each sample size in ADNI and OASIS. The shaded regions represent the variance across the 1,000 bootstrap replications. ICC values exceeding 0.75 indicate good reliability. ICC, Intraclass correlation coefficients.

**Table S1. Baseline characteristics of APOE4 non-Carriers and APOE4 carriers from the PPMI cohort**

| **Group** | **APOE4 non-Carriers** | **APOE4 carriers** |
| --- | --- | --- |
| n | 98 | 27 |
| Age, years | 61.84 (7.29) | 57.81 (6.24) |
| Sex, male/female | 36 / 62 | 13 / 14 |
| Education, years | 16.00 (2) | 16.00 (1.12) |
| CSF Aβ_42_, pg/mL | 1054.50 (313.75) | 974.10 (173.53) |
| **Cognitive performance** | | |
| MoCA | 28.00 (1) | 28.00 (1.12) |
| JLO | 12.80 (1.56) | 13.26 (1.4) |
| HVLT-R | 51.00 (7) | 52.00 (5.25) |
| Semantic Fluency | 51.50 (6.5) | 54.00 (9.5) |
| LNS | 12.00 (1.5) | 12.00 (1.25) |
| SDMT | 51.62 (7.5) | 50.00 (4.02) |

All values represent baseline data. Data are presented as median (interquartile range), except for sex, which is shown as the number of males and females. No significant differences were observed between APOE4 non-carriers and APOE4 carriers on any demographic, clinical, or cognitive variables. Group comparisons were performed using the Mann-Whitney U test for continuous variables and the Chi-square test for categorical variables. MoCA, Global cognition was evaluated with the Montreal Cognitive Assessment; JLO, Benton’s Judgment of Line Orientation; HVLT-R, the Hopkins Verbal Learning Test-Revised; LNS, the Letter-Number Sequencing test; SDMT, Symbol Digit Modalities Test.

**Table S2. Significant longitudinal associations between brain measures and cognitive domains in ADNI.**

| **Measure** | **Brain similarity** | | **Cortical thickness** | | **Hippocampal volume** | | **Ventricular volume** | | **Total GM volume** | |
| --- | --- | --- | --- | --- | --- | --- | --- | --- | --- | --- |
| **Outcome** | **R^2^** | ***P*** | **R^2^** | ***P*** | **R^2^** | ***P*** | **R^2^** | ***P*** | **R^2^** | ***P*** |
| **Incident Aβ-positive group** | | | | | | | | | | |
| Executive | 0.72 | <.001 | - | - | - | - | - | - | - | - |
| Attention | - | - | - | - | - | - | - | - | - | - |
| Memory | - | - | - | - | - | - | - | - | - | - |
| Language | - | - | - | - | - | - | - | - | - | - |
| Visuo spatial | 0.48 (0.07) | <.001 | 0.45 (0.06) | <.001 | - | - | - | - | - | - |
| PACC | 0.88 (0.02) | .019 | 0.85 (0.01) | .005 | - | - | 0.82 | .0214 | 0.81 | .0151 |
| ADL | 0.88 (0.04) | .003 | 0.79 (0.03) | .019 | - | - | - | - | - | - |
| **Elevated Aβ-positive group** | | | | | | | | | | |
| Executive | 0.7 (0.02) | <.001 | - | - | - | - | - | - | - | - |
| Attention | 0.78 (0.01) | <.001 | 0.76 (0.01) | .013 | - | - | - | - | - | - |
| Memory | 0.69 (0.01) | <.001 | 0.68 (0.01) | .002 | - | - | - | - | - | - |
| Language | 0.86 | <.001 | - | - | 0.84 | .0022 | - | - | - | - |
| Visuo spatial | - | - | - | - | - | - | - | - | - | - |
| PACC | 0.79 (0.01) | .001 | 0.75 | <.001 | 0.74 | .0111 | - | - | 0.74 | .03 |
| ADL | 0.41 (0.08) | <.001 | - | - | - | - | - | - | - | - |

Values represent R² and False Discovery Rate (FDR)-corrected *p* values derived from linear mixed-effects models examining the association between brain measure and longitudinal cognitive change. In all models, *p* values correspond to the interaction term between the predictor and time. For brain similarity and cortical thickness, which comprised multiple predictors, each predictor was modeled separately. In cases with multiple significant results, the median and interquartile range (IQR) of the R^2^ values are reported, the *p* value corresponds to the largest (i.e., least significant) among them. For single significant results, the R² and *p* value are reported. “-” indicates no statistically significant result after FDR correction. PACC, Preclinical Alzheimer’s Cognitive Composite; ADL, Activities of Daily Living Questionnaire; A, amyloid; R^2^, Coefficient of determination; *P*, *p* value; GM, gray matter.

**Table S3. Significant longitudinal associations between brain measures and cognitive domains in OASIS.**

| **Measure** | **Brain similarity** | | **Cortical thickness** | | **Hippocampal volume** | | **Ventricular volume** | | **Total GM volume** | |
| --- | --- | --- | --- | --- | --- | --- | --- | --- | --- | --- |
| **Outcome** | **R^2^** | ***P*** | **R^2^** | ***P*** | **R^2^** | ***P*** | **R^2^** | ***P*** | **R^2^** | ***P*** |
| **Incident Aβ-positive group** | | | | | | | | | | |
| Executive | 0.77 (0.02) | <.001 | 0.72 (0.02) | .005 | 0.68 | .014 | 0.69 | .002 | - | - |
| Attention | 0.52 (0.04) | .001 | 0.47 (0.04) | .01 | - | - | 0.39 | .004 | - | - |
| Memory | 0.61 (0.06) | <.001 | - | - | - | - | 0.48 | .005 | - | - |
| Language | 0.92 | <.001 | - | - | - | - | - | - | - | - |
| PACC | 0.72 (0.05) | <.001 | 0.66 (0.06) | .003 | - | - | 0.62 | <.001 | - | - |
| ADL |  |  |  |  |  |  |  |  |  |  |
| **Elevated Aβ-positive group** | | | | | | | | | | |
| Executive | 0.77 (0.01) | <.001 | - | - | - | - | - | - | - | - |
| Attention | - | - | - | - | - | - | - | - | - | - |
| Memory | 0.67 (0.04) | .007 | - | - | - | - | - | - | - | - |
| Language | 0.87 (0.01) | <.001 | - | - | - | - | - | - | - | - |
| PACC | 0.69 (0.03) | .009 | 0.65 (0.02) | .02 | - | - | 0.61 | .0128 | - | - |
| ADL | 0.41 (0.03) | .002 | 0.38 (0.03) | .001 | - | - | - | - | - | - |

Values represent R² and False Discovery Rate (FDR)-corrected p values derived from linear mixed-effects models examining the association between brain measure and longitudinal cognitive change. In all models, *p* values correspond to the interaction term between the predictor and time. For brain similarity and cortical thickness, which comprised multiple predictors, each predictor was modeled separately. In cases with multiple significant results, the median and interquartile range (IQR) of the R^2^ values are reported, the p value corresponds to the largest (i.e., least significant) among them. For single significant results, the R² and *p* value are reported. “-” indicates no statistically significant result after FDR correction. PACC, Preclinical Alzheimer’s Cognitive Composite; ADL, Activities of Daily Living Questionnaire; A, amyloid; R^2^, Coefficient of determination; *P*, *p* value.

**Table S4. Significant longitudinal associations between baseline measures and cognitive domains in ADNI.**

| **Dataset** | **CSF subset** | | | | | **Plasma subset** | | | | |
| --- | --- | --- | --- | --- | --- | --- | --- | --- | --- | --- |
| **Measure** | **Brain Similarity** | **P-tau181** | | **T-tau** | | **Brain Similarity** | **P-tau181** | | **NfL** | |
| **Outcome** | **R^2^** | **R^2^** | ***P*** | **R^2^** | ***P*** | **R^2^** | **R^2^** | ***P*** | **R^2^** | ***P*** |
| **Incident Aβ-positive group** | | | | | | | | | | |
| Executive | 0.72 | - | - | - | - | 0.72 | - | - | - | - |
| Attention | - | - | - | - | - | - | - | - | - | - |
| Memory | - | - | - | - | - | - | - | - | - | - |
| Language | - | - | - | - | - | - | - | - | - | - |
| Visuo spatial | 0.87 (0.01) | 0.86 | .018 | 0.87 | .013 | 0.48 (0.07) | 0.26 | .025 | - | - |
| PACC | 0.95 (0.01) | 0.93 | .012 | 0.93 | .006 | 0.86 (0.03) | 0.78 | .007 | 0.77 | .024 |
| ADL | 0.89 (0.09) | 0.82 | <.001 | 0.79 | .005 | 0.88 (0.04) | - | - | - | - |
| **Elevated Aβ-positive group** | | | | | | | | | | |
| Executive | 0.71 (0.02) | - | - | - | - | 0.71 (0.02) | - | - | - | - |
| Attention | 0.85 (0.02) | - | - | 0.82 | .018 | 0.79 (0.01) | - | - | 0.76 | <.001 |
| Memory | 0.71 (0.01) | - | - | - | - | 0.71 (0.01) | - | - | - | - |
| Language | 0.86 | - | - | - | - | 0.86 | - | - | 0.85 | <.001 |
| Visuo spatial | - | - | - | - | - | - | - | - | - | - |
| PACC | 0.79 (0.01) | - | - | - | - | 0.83 (0.01) | 0.78 | .001 | 0.79 | .004 |
| ADL | 0.41 (0.08) | - | - | - | - | 0.41 (0.08) | - | - | - | - |

Values represent R^2^ and False Discovery Rate (FDR)-corrected *p* values from linear mixed-effects models examining the association between baseline brain measures and longitudinal cognitive change. In all models, *p* values correspond to the interaction term between the predictor and time. Brain similarity included multiple predictors modeled separately and previously identified as significant in longitudinal analyses (Table S1), such that the table summarizes baseline model fit and omits *p* values. For brain similarity, when multiple significant results are present, the median and interquartile range (IQR) of the R^2^ values are reported; when only a single result is significant, only the R^2^ is provided. For biomarkers, both R^2^ and *p* value are reported. “-” indicates no statistically significant result after FDR correction. Subset details are provided in Table 1, and statistical methods for biomarkers are described in Section 2.6 of the main text. PACC, Preclinical Alzheimer’s Cognitive Composite; ADL, Activities of Daily Living Questionnaire; A, amyloid; R^2^, Coefficient of determination; *P*, *p* value; P-tau, tau hyperphosphorylation; T-tau, total tau; NfL, neurofilament light.

**References**

[1] Marek K, Jennings D, Lasch S, Siderowf A, Tanner C, Simuni T, et al. The Parkinson Progression Marker Initiative (PPMI). Prog Neurobiol 2011;95:629–35. https://doi.org/10.1016/j.pneurobio.2011.09.005.

[2] Pereira JB, Aarsland D, Ginestet CE, Lebedev AV, Wahlund L-O, Simmons A, et al. Aberrant cerebral network topology and mild cognitive impairment in early Parkinson’s disease. Hum Brain Mapp 2015;36:2980–95. https://doi.org/10.1002/hbm.22822.

[3] Weinshel S, Irwin DJ, Zhang P, Weintraub D, Shaw LM, Siderowf A, et al. Appropriateness of Applying Cerebrospinal Fluid Biomarker Cutoffs from Alzheimer’s Disease to Parkinson’s Disease. J Park Dis 2022;12:1155–67. https://doi.org/10.3233/JPD-212989.
